# Clinical electrophysiology of the optic nerve and retinal ganglion cells

**DOI:** 10.1038/s41433-021-01614-x

**Published:** 2021-06-11

**Authors:** Oliver R. Marmoy, Suresh Viswanathan

**Affiliations:** 1 Clinical and Academic Department of Ophthalmology, Great Ormond Street Hospital for Children, London, UK; 2 UCL-GOS Institute for Child Health, University College London, London, UK; 3 Manchester Metropolitan University, Manchester, UK; 4 College of Optometry, State University of New York, New York, NY USA

**Keywords:** Optic nerve diseases, Retina

## Abstract

Clinical electrophysiological assessment of optic nerve and retinal ganglion cell function can be performed using the Pattern Electroretinogram (PERG), Visual Evoked Potential (VEP) and the Photopic Negative Response (PhNR) amongst other more specialised techniques. In this review, we describe these electrophysiological techniques and their application in diseases affecting the optic nerve and retinal ganglion cells with the exception of glaucoma. The disease groups discussed include hereditary, compressive, toxic/nutritional, traumatic, vascular, inflammatory and intracranial causes for optic nerve or retinal ganglion cell dysfunction. The benefits of objective, electrophysiological measurement of the retinal ganglion cells and optic nerve are discussed, as are their applications in clinical diagnosis of disease, determining prognosis, monitoring progression and response to novel therapies.

## 摘要

使用图形视网膜电流图 (Pattern Electroretinogram, PERG)、视觉诱发电位 (Visual Evoked Potential, VEP) 和感光阴性反应 (Photopic Negative Response, PhNR) 以及其他更专业的技术可以进行视神经和视网膜神经节细胞功能的临床电生理评估。本文中, 我们描述了这些电生理技术及其在除青光眼以外的影响视神经和视网膜神经节细胞疾病中的应用。讨论的疾病组包括遗传性、应力性、毒性/营养性、外伤性、血管性、炎性和颅内原因导致的视神经或视网膜神经节细胞功能障碍。本文讨论了客观的、电生理测量视网膜神经节细胞和视神经的优势, 以及它们在疾病的临床诊断、评估预后、监测进展和对新疗法的反应等方面的应用。

## Introduction

The optic nerve and retinal ganglion cells (RGCs) are essential in the transmission of visual information through the intracranial pathway to the striate/primary visual (V1) cortex. Diseases of the optic nerve and RGCs therefore can lead to significant visual impairment and may be a primary pathology or secondary consequence of other conditions. Whilst ophthalmic imaging and psychophysical tests can provide insight into structural and behavioural sequelae of optic nerve dysfunction, functional assessments through electrophysiology provide an objective and quantitative approach to characterise these deficits directly at the level of optic nerve and RGCs.

The electrophysiology of the optic nerve and RGCs has been well established through the Visual Evoked Potential (VEP) and Pattern Electroretinogram (PERG), and more recently with the Photopic Negative Response (PhNR) alongside other specialised techniques like the multifocal electroretinogram, PERG and VEP (mfERG, mfPERG, mfVEP) to provide a more detailed evaluation of the retinal locus and spatial extent of cellular dysfunction. These prospects are promising in the phenotyping and characterisation of optic nerve disease where other clinical information may not provide sufficient information.

In this review, we discuss the electrophysiological basis of optic nerve and RGC disease and its role in investigating the site and extent of dysfunction to complement structural and psychophysical findings in disease. We start by defining our search strategy, followed by a description of the main electrophysiological techniques used to assess the optic nerve and RGCs, including their stimulus and recording parameters. We then discuss the clinical applications of these techniques to diseases or conditions affecting the optic nerve and RGCs, lastly concluding with diagnostic aids and dilemmas commonly encountered in ophthalmic and neuro-ophthalmic practice.

## Methods

A comprehensive search of literature on Medline (PubMed), ScienceDirect, the Cochrane Library was performed by the authors. Search terms included respective diagnostic tests and their related terms or abbreviations (i.e. Visual* evoked potential OR VEP OR Visual* evoked response) against the clinical condition of interest (i.e. Optic neuritis OR demyelin* OR papillitis OR multiple sclerosis). Broad search terms were used to capture a wide range of literature. In circumstances where standard search terms retrieved few results, search terms were broadened to include more general electrophysiological terms (i.e. electrophysio* OR electroret* or electrodiag*). No limits were applied in relation to publication dates but only articles available in the English language were reviewed. Each article had a full-text review and was critically appraised by the authors. Additional review of references within qualifying publications was also undertaken in search of any further published works relevant to this review. The main interests to this review were studies employing electrophysiological techniques within the main clinical conditions, from which the authors used their clinical experience to determine the most relevant and clinically useful findings to this review. Animal studies were generally excluded unless of particular importance to underpinning science or methodology.

## Electrophysiological tests of optic nerve and retinal ganglion cell function

### Visual evoked potential (VEP)

The VEP is an important clinical test for assessing the functional integrity of the visual pathway from the retina to the striate cortex *(primary visual cortex or V1)*. As such, this test has been extensively used in the evaluation of ophthalmic, neurological and systemic disease. The VEP is produced from activation of cortical neurons in response to afferent pathway stimulation, which is recorded with electrodes placed over the occiput. Typically, VEPs are recorded to a high contrast pattern or diffuse flash stimuli. For pattern VEPs, a checkerboard or grating stimulus is reversed in contrast over time whilst maintaining a constant mean luminance (PR-VEP), or alternatively the checkerboard appears and disappears on a background with the same mean luminance known as the pattern onset-offset VEP (PO-VEP), which provide information regarding the function of the macular pathways. Flash VEPs (F-VEP) are typically recorded to a strobe or LED flash stimulator and is useful in the examination of the generalised visual pathway function, particularly in eyes with poor optical quality where retinal image contrast is degraded.

**Fig. 1:**
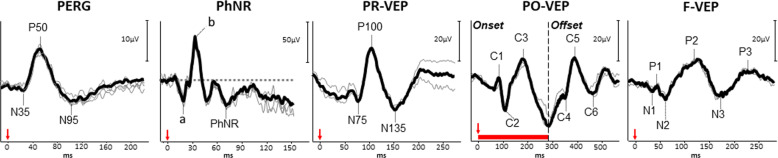
Illustrative waveform examples for the major electrophysiological tests in the assessment of optic nerve and retinal ganglion cell (RGC) function. In all panels, relative amplitudes are observed in the top right, with timings seen on the *X*-axis. The left most panel shows the transient pattern electroretinogram (PERG), following the stimulus (red arrow) an initial N35 negativity is seen followed by the main positivity (P50) and large later negativity (N95). The photopic negative response (PhNR) is next seen, recorded to a diffuse flash stimulus (red arrow). The a- and b-waves of the ERG are seen of the typical flash ERG, followed by the late negativity known as the PhNR. In the central panel the pattern reversal VEP (PR-VEP) is seen, a triphasic response with N75, P100 and N135 peaks respectively. The pattern onset-offset VEP (PO-VEP) response is seen to a longer stimulus (red bar), the initial response comprises the onset VEP of C1-C3 components, later followed by the offset VEP C4-C6 components. Lastly the flash VEP (F-VEP) is seen to a diffuse flash stimulus (red arrow), followed by a series of positive-negative deflections, with the major N2 and P2 peaks observed.

The typical PR-VEP waveform comprises a triphasic response, with components named according to their relative polarity and peak-time. A major positive peak is seen around 100 ms (P100), with preceding and following negativities around 75 ms (N75) and 135 ms (N135) respectively (Fig. 1). The PO-VEP waveform is more complex, with onset C1, C2 and C3 components which are more variable between individuals and change in shape during the normal lifespan [1], with offset responses typically demonstrating a triphasic waveform similar to the PR-VEP (Fig. 1). The F-VEP waveform is also complex comprising of multiple peaks and troughs named N1, P1, N2, P2, N3 and P3 respectively. The major positive peak (P2) and preceding negativity (N2) are most commonly used in clinical assessment as these waveforms are most robust. The interpretation of responses is performed by assessing the response amplitude (from the preceding negativity or baseline), peak-time/latency, morphology and transoccipital distribution. The amplitude and latency of responses should be compared to reference values, which are collected or validated to the local laboratory environment as with all clinical visual electrophysiology techniques. Generally, different conditions of the optic nerve or RGCs may affect response amplitude or latency preferentially, the VEP has a large macular contribution and can be affected by anything upstream in the visual pathway, for example a maculopathy may degrade responses as well as primary RGC disease. Abnormalities of the VEP should therefore always be explored with the PERG to elaborate on the site and extent of dysfunction.

There are regularly reviewed international standards for performing clinical VEPs [2]. Whilst these standards provide the minimum recording requirements for performing a VEP, it is emphasised that these are a set of minimum standards and those performing VEPs should expand on these standards with additional protocols to address more complex clinical questions [3]. Interpretation of VEPs should rarely be used in isolation as this could lead to misdiagnosis. For example, even in suspected neurological dysfunction an abnormality of a P-VEP is not specific to optic nerve disease, as the response is subject to good macular integrity and therefore assesses the visual pathway from the macula to striate cortex and should be explored with the PERG where abnormal.

The PR-VEP has relatively low inter- and intra-subject variability which has facilitated its clinical use, whereas the PO-VEP and F-VEP can be more variable between individuals and therefore limits their clinical applications [4–7]. Nevertheless, these stimuli do have benefits in the assessment of patients with poor vision, low cooperation, detection of intracranial pathway dysfunction and inter-ocular differences. PR-VEPs are largely dominated by input from the macula, with expanded representation of the central field, which is scaled topographically known as cortical magnification [8, 9]. As such, the PR-VEP shows a U-shaped spatial tuning function against check width, alongside being modulated by stimulus luminance, contrast and field size [10]. These properties can be used to a great advantage in some circumstances to exceed ISCEV recording standards, for example recording PR-VEPs to a range of check widths or to reduced stimulus contrast can increase diagnostic sensitivity, with VEP abnormalities sometimes preceding structural change in optic nerve dysfunction, which will be discussed in more detail later in this review [11–15].

It should also be noted that the pattern VEP in clinical practice is typically recorded to an achromatic pattern stimulus, but it is possible to use chromatic stimuli to assess parvocellular and koniocellular parallel pathways of the visual system using red-green or blue-yellow stimuli respectively. Chromatic VEPs have been used to identify colour processing dysfunction in demyelinating disease [16–18], LHON [19], Glaucoma [20], Parkinsons disease [21] and congenital colour blindness [22]. Their use is intriguing in conditions of the optic nerve and RGCs causing dyschromatopsia or more selective RGC pathway deficits. However, the stimuli required for testing are technically challenging to achieve, requiring reproducible spectral stimulus properties, isoluminance, alongside age- and ethnicity-controlled reference data to allow for macula pigment and the effects on cone fundamentals which has overall limited their widespread clinical use [23].

### Pattern electroretinogram (PERG)

The PERG provides information regarding macula and RGC function and thus has a role in the investigation of both ophthalmic and neurological disease. The PERG is able to delineate the site of dysfunction in those patients with abnormal PVEPs, quantify the extent of dysfunction, and to provide spatial information regarding the functional visual field. The transient PERG is typically produced to a reversing checkerboard or grating stimulus with a constant mean luminance presented at ~4 rev/sec, with the generated response comprised of a sometimes ill-defined small initial negativity (N35), followed by a major positivity around 50 ms (P50) and large later negativity around 95 ms (N95) (Fig. 1). The P50 component has contributions from both the outer- as well as inner-retinal neurons, including the RGCs. Conversely, the N95 component is solely generated by spiking activity of RGCs and is sensitive to retinal nerve fibre degeneration and RGC loss [24, 25].

The PERG is a small signal and typically recorded with corneal electrodes, which do not affect the visual optics. Skin electrodes are not routinely used due to the poor signal-to-noise ratio, but do have advantages in those unable to tolerate corneal electrodes such as children [26]. Since the PERG is elicited in response to contrast modulation of a pattern stimuli, optimal refractive correction is necessary to ensure that the retinal image contrast is not degraded. The PERG is more sensitive to contrast changes and defocus than the PVEP, likely due to the PERG being a reflection of direct retinal activity to pattern stimuli, whereas for the PVEP several post-retinal processes occur which have some modulation and compensation for low contrast and defocus, alongside cortical magnification, making the PVEP more robust than the PERG to these changes. The PERG is typically recorded to a standard 15° field size, but an additional large field of 30° may also be used which can provide further topographic information of paramacular function [27]. Furthermore, simultaneous recording alongside a PR-VEP allows control for fixation and defocus, therefore it can be useful in patients with functional visual loss. The PERG to high temporal frequencies (typically >10rev/sec) generates a steady-state response, which is not widely used in clinical practice but does have some clinical applications in conditions like Glaucoma (*reviewed elsewhere within this issue*). As with the VEP, there are international minimum standards for recording the clinical PERG [28].

The interpretation of the PERG provides assessment of the overall outer retinal and RGC/optic nerve pertaining to the macula. A reduction in amplitude of the P50 component with normal or slightly delayed peak-time and preservation of the N95:P50 ratio is reflects dysfunction anterior to the RGCs (i.e. cone photoreceptors or bipolar cells) or reduced retinal image contrast. Conversely, a predominant reduction in the N95 amplitude with an intact P50, which reduces the N95:P50 ratio, is characteristic of RGC and optic nerve dysfunction (Fig. 1)[25]. Importantly however, in severe RGC disease there can be reduction in P50 amplitude and shortening of peak-time, as the P50 component has some contributions from RGCs [24]. The P50 component should therefore not be extinguished unless there is concomitant dysfunction anterior to the RGCs (i.e. from a maculopathy). A normal PERG but abnormal PR-VEP localises dysfunction to outside of the central RGCs or posteriorly along the visual pathway.

### Photopic negative response (PhNR)

**Fig. 2:**
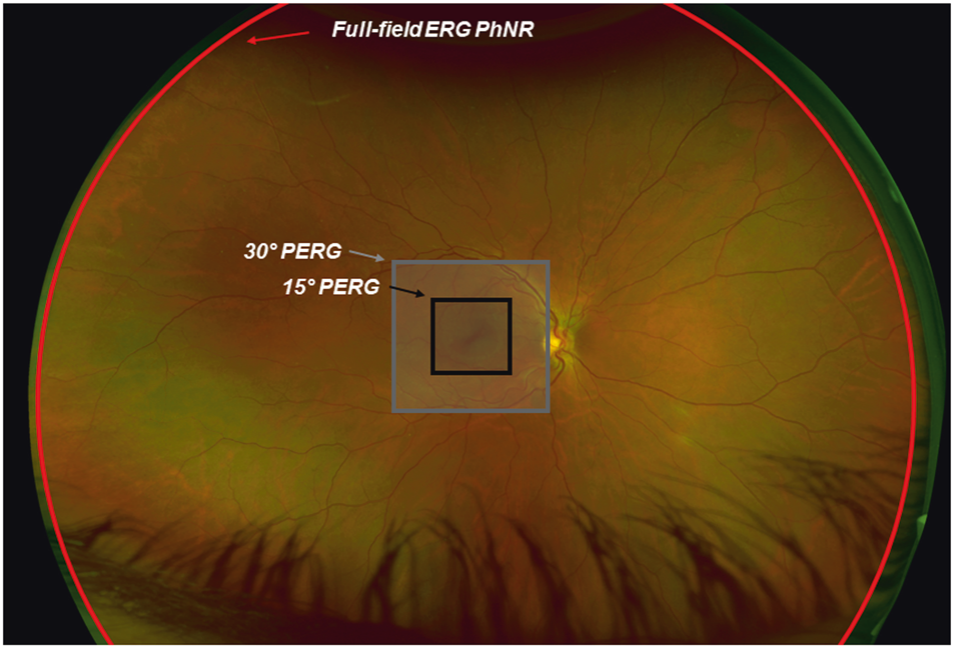
Stimulus conditions for the pattern ERG (PERG) and Photopic Negative Response (PhNR) relative to the retinal topography from an optomap image. This figure illustrates the full-field nature of the PhNR (red circle) being a pan-retinal response, whereas the PERG is recorded to a central stimulus subtending 30° (grey box) or 15° (black box) of the central retina respectively.

The PhNR is a slow negative potential that follows the *b*-wave of the light-adapted flash ERG and originates as a consequence of spiking activity of inner-retinal neurons, predominantly the RGCs [29]. While the PhNR can be elicited with the ERG stimuli used in standard clinical testing [30], the optimal stimulus that elicits the maximal amplitude reduction in RGC dysfunction is a red flash on a rod-saturating blue background [31, 32]. The PhNR amplitude is typically measured at its trough (or at a fixed time in the range of 65–75 ms after the onset of the test flash) from the baseline [33] (Fig. 1). Other methods of evaluating the PhNR amplitude includes measurement from the trough to the peak of the preceding b-wave or expressing this value as a ratio of the b-wave amplitude [29, 34–36]. The PhNR is typically elicited with a full-field flash stimuli and consequently reflects the summed activity of RGCs across the entire retina and cannot determine the spatial localisation of the RGC defect. Typical recording parameters are included in the ISCEV extended protocol [33]. However, having the responses of retinal bipolar cells and cone photoreceptors within the same ERG waveform is useful to discern the functionality of the neurons that provide input to the RGCs. In addition, since the visual stimulus is diffuse, the responses are not affected by optical defocus and thus refractive correction is not required, furthermore this may aid in the identifying generalised RGC loss across the retina while the PERG is normally limited to the macula (Fig. 2).

### Multifocal techniques

The mfERG employs computational techniques that allow obtaining sub-microvolt focal ERG responses simultaneously from multiple retinal areas within a relatively short recording time [37]. The mfERG responses are organised in kernels with responses increasing in their degree of complexity with increasing kernel orders. The first-order kernel responses that are typically analysed with standard clinical mfERG testing [38] does not contain obvious RGC responses. However, more sophisticated algorithms allow the separation of an Optic Nerve Head Component from the first-order kernel responses which in turn is abolished in eyes with optic nerve abnormalities [39]. Variations from standard mfERG stimulation techniques can allow recording PhNRs from discrete retinal regions [40, 41].

The multifocal recording technique can be combined with the contrast reversal of pattern stimulus elements, scaled in size across the visual field to reflect the cortical magnification factor, to enable recording focal VEP responses from the visual cortex [42, 43]. This technique is commonly referred to as the multifocal VEP (mfVEP) and allows topographical analysis of the VEP through the detection of regional changes in amplitude and waveform delay, that may not be reflected in the gross potentials measured with conventional VEP recording.

Similarly, mfERG recordings to a contrast reversal of pattern stimuli from discrete retinal regions termed the multifocal PERG (mfPERG) demonstrate second order kernel responses with the waveform shape resembling that of the conventional transient PERG for slower stimulation sequences [44]. There is compelling evidence to support the notion that the mfPERG response originates from activity of inner-retinal neurons and is likely generated at the optic nerve head [45, 46]. While multiple studies have demonstrated the reduction of mfPERG amplitude in several disease conditions, its ability to detect focal defects as opposed to a generalised reduction may be disease specific [45, 47–52].

### Other methods

Neuronal contributions to the full-field flash ERG can also be elicited under dark-adapted conditions. The Scotopic Threshold Response (STR) is an ERG response with negative polarity and a slower time-course than the PhNR that can be elicited with very dim flashes of light, close to the absolute visual threshold under strictly dark-adapted conditions [53, 54]. The STR reflects a combination of RGC and amacrine cell responses mediated by the rod pathway. This ERG measure has not gained clinical utility mainly due to dark-adaptation requirements.

The oscillatory potentials (OPs) are high frequency responses predominantly of amacrine cell origin [55, 56] typically measured in the range of 70–300 Hz in the standard clinical ERG [30]. The OPs manifest as wavelets on the ascending limb of the b-wave of the flash ERG that can be seen for ERG measurements under both dark- and light-adapted conditions. While the OPs are generally associated with amacrine cells, the oscillations themselves are considered to be modulations of bipolar cell responses by inhibitory feedback of amacrine cells [57] and is not further discussed in this review.

## Clinical applications

### Hereditary optic neuropathies and primary retinal ganglion cell disease

Autosomal Dominant Optic Atrophy (ADOA) is a degenerative condition of the optic nerve that affects both eyes and is characterised by gradual deterioration of vision, typically starting in early childhood [58–60]. Mutation of the OPA1, OPA3, genes that code for proteins associated with electron transport and ATP synthesis in the mitochondrial inner membrane and three loci (OPA4, OPA5, OPA8) underlie the genetic defect in ADOA [58]. In addition, defects with other loci like OPA2, OPA6 and OPA7 genes underlie x-linked and recessive inheritance of optic atrophy [58].

The severity of visual impairment is variable from a mild reduction in visual acuity to blindness and around 20% of patients with ADOA also manifest neurological symptoms outside the visual system [58]. ADOA patients develop visual field defects that typically arise as central or centrocecal scotomas, with commensurate peripapillary RNFL thinning on OCT measurements alongside optic disc pallor [61]. Visual rehabilitation with low vision aids and genetic counselling currently remain the focus for management of this condition [61], although there is promise in animal models for gene therapy [62].

Visual electrodiagnostic techniques complement the behavioural assessment of visual function in ADOA. Specifically, it pinpoints the functional deficit to the level of the RGCs and the optic nerve and is also quite useful as a reliable objective measure of visual function when behavioural testing is unreliable, for example in children. PR-VEP waveforms are delayed with reduced amplitude in ADOA patients with mild to moderate severity and can be extinguished in advanced cases [63–65]. Furthermore, the waveform may become bifid or of a ‘p-n-p’ morphology due to enhancement of paramacular PR-VEP components which can occur with reduced central visual field sensitivity [66–68]. The N95 component of the PERG is reduced but the P50 component is mostly normal or reduced in severe cases where the N95 is extinguished. Analysis of the PR-VEP changes in combination with the alteration of the N95 and P50 components of the PERG recordings can confirm the locus of the functional deficit in ADOA to the level of RGCs and the optic nerve. The PhNR of the flash ERG is reduced in ADOA with sparing of the a- and b-waves, reiterating the RGCs as the locus of the functional deficit [69, 70]. While the PhNR for both full-field and focal flash ERG recordings are reduced in ADOA, the PhNR recorded to focal stimulation demonstrates a higher sensitivity for detection of ADOA, approaching the sensitivity estimates of the PERG [70]. These findings highlight the importance of using focal stimulation for optimal electrophysiological testing of ADOA patients who typically demonstrate central visual field defects.

Leber’s hereditary optic neuropathy (LHON) is another condition characterised by the selective degeneration of RGCs due to mutations associated with genes encoding for mitochondrial proteins involved in oxidative phosphorylation [71]. In majority of the cases, the genetic defect in LHON is associated with the mutation of mitochondrial DNA 11778G>A, 14484T>C and 3460G>A [72]. The condition is characterised by bilateral painless reduction in visual acuity with development of centrocecal scotoma resulting from papillomacular nerve fibre bundle degeneration and is typically seen in men in the second or third decade of life [60, 61]. Retinal and circumpapillary telangiectasia, increases in RNFL thickness and pseudo-oedema of the optic disc can be observed prior to symptomatic vision loss [73–75]. Optic disc pallor can be observed subsequently starting with the inferotemporal quadrant with progressive RNFL thinning [75]. While visual prognosis was generally considered to be poor in symptomatic LHON cases, newer treatments such as Idebenone have been demonstrated to improve visual outcomes through stimulation of ATP synthesis and free radical scavenging in mitochondria [76]. Further, at the time of writing early studies of gene therapy appear promising in humans [77, 78].

**Fig. 3:**
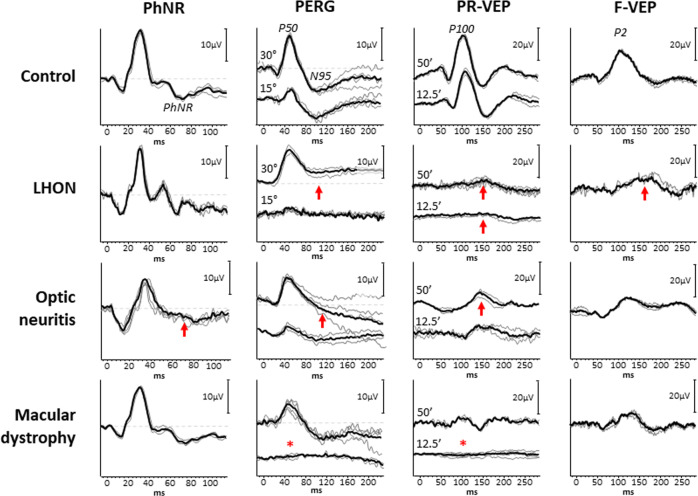
Illustrative case series demonstrating the electrophysiological findings in a healthy subject, and affected eyes of patients with Lebers Hereditary Optic Neuropathy, Optic Neuritis and Macular dystrophy respectively. The top row is used for reference, to demonstrate the normal findings of the photopic negative response (PhNR), pattern electroretinogram (PERG) (to 30° and 15° fields), pattern reversal visual evoked potential (PR-VEP) (to 50′ and 12.5′ check widths) and flash visual evoked potential (F-VEP). *LHON:* The PERG demonstrates normal P50 components but markedly abnormal N95 components which do not fall below the baseline, to the 15° field the P50 is also reduced with early peak-time. The PR-VEP is severely degraded to both check widths, with only a small response to large check widths, with the F-VEP broad and slightly low amplitude but preserved. The PhNR is relatively preserved, although does not fall below the a-wave. Overall, this indicates marked bilateral retinal ganglion cell (RGC) and optic nerve dysfunction, with some preservation of peripheral RGC function. *Optic neuritis:* The PERG shows normal P50 components but mildly reduced N95 components to both field sizes. The PR-VEP is atypically delayed but with preserved amplitude, alongside the F-VEP which is preserved. The PhNR is markedly reduced with normal a- and b-waves. Overall, this indicates some optic nerve dysfunction with some level of retrograde degeneration to the RGCs centrally, with marked peripheral RGC dysfunction. *Macular dystrophy:* The PERG P50 to a 30° field is well defined, but that to a 15° field is absent indicating marked macular dysfunction localised to the central 15° field. The PR-VEP to 50′ check widths is of normal peak-time but borderline amplitude, with loss of the PR-VEP to small check widths. The F-VEP is of appropriate amplitude, with the PhNR within normal limits also. Overall, this indicates localised macular dysfunction affecting the 15° field with preservation of the surrounding 15–30° field. The preserved N95:P50 ratio and PhNR indicating normal RGC and optic nerve function.

In LHON cases with clinically distinct optic atrophy the P100 component of the PR-VEP, if not extinguished, is delayed with reduced amplitude and the PERG demonstrates reduced amplitude of the N95 component [79–81]. PR-VEP and PERG abnormalities can manifest in affected eyes prior to clinically visible temporal pallor of the optic disc, with PR-VEP alterations being more prevalent than PERG alterations [81]. Interestingly, the amplitude of the N95 component of the PERG is selectively reduced in eyes of some patients classified as LHON carriers, based on incomplete penetrance in the absence of VEP abnormalities, leading to question whether the PERG amplitude reduction represents subclinical changes and could serve as a marker for those who may convert to the acute phase [80]. Indications of subclinical changes in some LHON carriers is also supported by abnormal findings with specialised psychophysical testing [19, 82]. The PhNR amplitude is also reduced in LHON patients with sparing of the a- and b-waves localising the functional deficit to the RGCs with measurements from baseline as opposed to the peak of the b-wave being more sensitive [19, 83, 84]. As with the PERG, the PhNR amplitude is also reduced in the eyes of some LHON carriers with the amplitudes showing a graded effect with RNFL thickness [83, 84]. In addition to its use in the assessment of RGC function at the time of diagnosis, visual electrophysiology techniques are also useful in assessing the efficacy new treatments for LHON [76]. An illustrative case of a patient with LHON is seen in Fig. 3.

There are several rare inherited disorders that include visual dysfunction where the PhNR is reduced and can assist in the simultaneous evaluation of optic nerve and retinal function. Hereditary Motor and Sensory Neuropathy Type VI is a condition where the PhNR is reduced. It is a severe form of Charcot-Marie-Tooth disease type 2A resulting from mutations in the MFN2 gene that codes for proteins in the outer mitochondrial membrane and the condition is characterised by peripheral axonal neuropathy with up to 20% of patients with the severe form of this condition also manifesting optic atrophy [85, 86]. Patients with late onset visual loss have a better chance of visual acuity improvement compared to patients with early onset of visual loss [85]. However, even patients with late onset visual loss who regain near normal visual acuity can demonstrate a severe reduction in the PhNR amplitude when the a- and b-waves are in the normal range suggesting the value of electrodiagnostic testing in understanding the full extent of the optic nerve functional deficit [87]. Interestingly, PR-VEP abnormalities have also been observed in Charcot-Marie Tooth disease of other genotypes, some without optic atrophy [88–91].

EAST syndrome is another genetic disorder where the PhNR amplitude is reduced [92]. This syndrome characterised by Epilepsy, Ataxia, Sensorineural deafness, and Tubulopathy resulting from a mutation of the KCNJ10 gene coding for Kir4.1, an inward rectifying potassium channels in the brain, inner ear and kidney [93]. These inward rectifying potassium channels are also expressed on retinal glial cells, which play a role in the siphoning and redistribution of potassium ions in the extracellular space [94]. A selective reduction in the PhNR amplitude in addition to providing confirmation of a deficit in retinal function in symptomatic patients reiterates previous hypotheses of the PhNR ERG potential also reflecting a glial mediated current secondary to extracellular potassium, resulting from RGC activity [29, 95]. Thus, in conditions such as EAST syndrome when the PhNR amplitude reduction can be prone to more than one interpretation, additional tests such as the PERG and VEP will be useful to assess the full extent of RGC and optic nerve function.

## Compressive, infiltrative, toxic and nutritional optic neuropathies

Compressive optic neuropathies or those secondary to space occupying lesions can cause significant disruption to optic nerve and RGC physiology. Intracranial tumours may affect any portion of the visual pathway and, as such, the VEP is well suited to provide assessment in localising the pathway lesion and information of pathway integrity. Whilst this review focuses on conditions affecting the optic nerve and RGC’s, it is prudent to discuss lesions affecting the entire visual pathway to the striate cortex which may later inflict dysfunction of the optic nerve or RGCs, for example due to retrograde degeneration of RGCs. As such, VEPs are a useful tool in the examination of the intracranial visual pathway especially when used in conjunction with the PERG and/or PhNR.

When utilising a transoccipital array of electrodes, one can use the lateralising features of VEP distributions to identify chiasmal and retrochiasmal pathway dysfunction. The lateralisation of the flash VEP has been demonstrated in both chiasmic and retrochiasmic lesions. Seminal studies using F-VEP in patients with homonymous field defects have demonstrated the major positive peak to become altered in lesions relating to the underlying field defect [96, 97]. As such, in lesions of the chiasm, one can observe a ‘crossed’ asymmetry of pattern or F-VEPs, whereby the transoccipital asymmetry will alter its lateralisation dependent on the eye stimulated [98]. These lateralising features have been adopted clinically to identify both chiasmal misrouting and a paucity of functional crossing fibres at the chiasm resulting from developmental or lesion related deficits of the chiasm [98–101]. The lateralising features of the VEP can be also used in retrochiasmic lesions using a flash or pattern stimulus to produce an ‘uncrossed’ asymmetry (i.e. a transoccipital asymmetry which remains static regardless of the eye stimulated) [102–106]. However, hemifield PR-VEPs are a far more advantageous method for investigating these intracranial visual pathway abnormalities [107]. One must be aware that, with a large stimulus field, large check size and a mid-frontal reference electrode, the paradoxical lateralisation phenomenon occurs with PR-VEPs, whereby the major positivity is observed over the occiput contralateral to the generating hemisphere [108]. This phenomenon is not observed in the pattern onset VEP, but has been observed in the pattern offset VEP [109]. Selective stimulation of the right- or left-hemifield can isolate the visual pathway contributions and allow localisation of the pathway dysfunction site, for example a bi-temporal hemifield loss in the PR-VEP would suggest chiasmal dysfunction, whereas a homonymous left hemifield loss would indicate a right hemisphere dysfunction. Whilst beyond the scope of this review, the benefits of multichannel VEPs in the investigation of intracranial pathway abnormalities are encouraged and are discussed elsewhere within this issue, particularly of benefits in patients unable to undertake visual field examination.

**Fig. 4:**
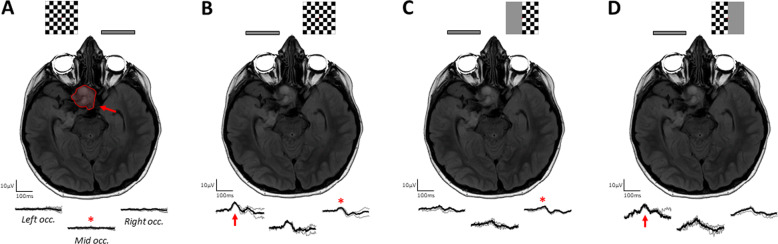
An illustrative case of compressive/infiltrative optic neuropathy in an 8 year old male patient with an optic pathway glioma involving the left optic nerve, chiasm and left optic tract (red arrow and outline). From the left, the MRI is shown and checkerboard stimuli demonstrating the presentation of the pattern stimulus respectively. The occipital VEP responses are shown at the bottom from the left, middle and right occiput respectively. **A** No reproducible PR-VEP is evident to left eye stimulation. **B** A reproducible PR-VEP is seen at the occiput to full-field PR-VEP stimulation of the right eye, however this is best defined over the left- and mid-occiput (red arrow) and attenuated over the right occiput (red asterisk). **C** Selective right half-field stimulation for the right eye demonstrates a reduced ipsilateral positivity expected (red asterisk). **D** Selective left half-field stimulation of the left eye demonstrates a preserved positivity (red arrow) similar to the full-field PR-VEP. Overall, PR-VEPs indicate profound macular pathway dysfunction affecting the LE and RE crossing fibres, but relatively preserved RE non-crossing fibres subserving the left half-field.

Compression of the anterior visual pathway can be from a variety of causes, for example optic pathway glioma, craniopharyngioma, haemangiomas, pituitary adenoma, meningiomas or cerebral aneurysms near the visual pathway which may exert pressure on the optic nerve, some of which being infiltrative. The pattern of visual loss in anterior visual pathway lesions can affect both eyes, for example where the chiasm or tracts are affected, or one eye, for example where the globe or optic nerve are unilaterally affected. Several studies of VEPs have investigated the pattern and flash VEP in compressive lesions of the anterior visual pathways [106, 107, 110–113], including intraoperatively during surgical decompression [114]. These studies overall demonstrate that in compressive lesions of the anterior visual pathway the PR-VEP will become delayed, with variable degrees of amplitude loss, alongside morphology changes in the waveform. Interestingly it has also been demonstrated that VEPs can be abnormal even in the absence of a measurable visual field defect or reduction in high contrast visual acuity [111, 115–117]. Furthermore, particularly for optic pathway gliomas, VEPs have high sensitivity for detecting functional abnormality [118, 119], which is particularly advantageous in children for whom behavioural perimetric testing is unreliable [120, 121] although their ability to monitor progression is less certain. Perhaps most relevant in compressive lesions are the aforementioned transoccipital distribution of VEPs, ideally using half-field stimuli, which is particularly important in the investigation of delayed VEPs, as chiasmal or retrochiasmal lesions are much less frequently observed in demyelinating disease [98, 122] (Fig. 4). The mfVEP has also been studied in compressive anterior pathway lesions and tends to show similar changes to behavioural perimetric findings [123–126], but are of debatable clinical use over existing techniques due to their demanding technical requirements. Interestingly, the mfPERG has been demonstrated to show good correlation between amplitude and degree of VF defect in chiasmatic lesions [49, 50].

The PERG in compressive lesions predominantly shows reduction in the N95 component, although P50 amplitude reduction is seen where PR-VEPs are severely abnormal [79]. It has also been demonstrated that PERG abnormalities are associated with worse visual outcome post-surgically, as this reflects retrograde degeneration to the RGCs rather than any transient or reversible visual loss [79, 127, 128]. The PhNR in compressive optic neuropathy was demonstrated in five patients where amplitude was found to be reduced similar to patients with AION [129]. Following this finding, a later study by Moon et al. [130] demonstrated in 18 patients with chiasmal compression that PhNR was reduced relative to controls but importantly that this correlated significantly with post-operative visual field sensitivity recovery, suggesting the PhNR has prognostic value. Moon et al. [131] also showed in their accompanying work that the PhNR reduction persisted and had some improvement by six months, albeit this did not reach statistical significance. The prognostic value of the PhNR against the PERG in predicting the recovery of visual function following treatment of compressive optic neuropathy is yet to be determined. The PhNR has also been reported to be significantly reduced in childhood optic pathway glioma, and demonstrated a strong relationship with the mean RNFL thickness [132]. These authors also remarked how the PhNR recording was achievable in all children, whereas OCT examination was not possible in one-third of their cohort due to motion artefacts or scan quality, which is a valuable concern for paediatric practice.

Endocrinological disorders of the thyroid, such as in Graves disease, can give rise to thyroid-associated orbitopathy (TAO). In TAO, the retro-orbital space can become reduced due to muscular fibrosis, inflammation and fat accumulation which causes compression of the optic nerve, known as dysthyroid optic neuropathy (DON). In DON, the PERG can show early optic nerve involvement [133, 134] with N95 amplitude loss, although P50 reductions have also been reported [134, 135]. VEP findings tend show latency delay and variable degree of amplitude reduction [134, 136–140], which can improve following surgical decompression [141]. Further to TAO, PR-VEPs have also been studied in autosomal recessive osteopetrosis, where latency delay can be seen [14]. In particular, PR-VEPs recorded to higher spatial frequencies (i.e. small check widths) in a range of compressive optic neuropathies may be more sensitive in detection of early changes associated with compression of the anterior visual pathway [14, 113, 120, 139].

PR-VEP amplitude reduction is a hallmark finding in cases of toxic optic neuropathy [142–145] and VEP responses can be subnormal even after visual acuity recovery suggesting persistence of subclinical changes [146, 147]. However, VEPs performed in isolation cannot localise the functional deficit to the level of the optic nerve and must be performed in conjunction with pattern and flash ERG to confirm RGC and/or optic nerve functional deficit with a normally functioning outer retina [148, 149].

VEP waveform delay and amplitude reduction is not a surprising finding in patients with traumatic optic neuropathies. However, interestingly there are multiple reports suggesting that VEP measurements obtained soon after traumatic optic nerve injury not only correlates with the patient’s visual acuity but may also have some predictive value towards visual prognosis, more so in patients unable to respond to subjective visual testing at the time of injury [150–155]. The value of the PERG and PhNR in assessing RGC function in traumatic optic neuropathy has seldom been explored.

VEP, PERG and PhNR have been employed extensively in studies of Glaucoma in the clinical setting for evaluation of RGC dysfunction and death and this information is covered elsewhere in this issue.

## Vascular causes of optic nerve and RGC dysfunction

Metabolic and vascular abnormalities secondary to hyperglycaemia contribute to diabetic neuropathies including diabetic retinopathy [156]. PR-VEP changes manifest as a combination of response delay and amplitude reduction, overall with response delays preceding amplitude delays in diabetics with either mild or no retinopathy [157–163]. PR-VEP changes are typically accompanied by brainstem auditory and somatosensory evoked potential abnormalities indicating that there may be a more diffuse deficit of the central sensory systems [164–167]. The PR-VEP abnormalities in diabetics generally tend to be accompanied by PERG changes, suggesting that the VEP changes to a large extent are likely a reflection of RGC dysfunction [161, 168, 169] and PERG changes are graded with the severity of diabetic retinopathy with demonstrable amplitude reduction, sometimes with this tendency seen also in patients with no visual symptoms or clinical signs of retinopathy [160, 168, 170, 171] and with interaction of factors like age of onset and duration of diabetes [170]. The b-wave of the dark-adapted flash ERG is affected with increase in duration and severity of diabetic retinopathy [160] thus warranting careful evaluation of photoreceptor and bipolar cell function with the flash ERG which also allows testing RGC function with the appropriate stimulus conditions. There is general consensus in the published literature that the PhNR amplitude is reduced in diabetic eyes and that there is a graded effect on the PhNR amplitude with increasing disease severity, with b-wave reduction accompanying PhNR changes in severe stages [171–175]. However, there is some disagreement on how early in the disease process the PhNR amplitude changes manifest [171, 173–175]. While some studies demonstrate a significant reduction or a tendency towards reduction of PhNR amplitude in eyes with no retinopathy [171, 173, 175], others report no change [172]. This discrepancy can be attributed to differences between these studies in the ERG stimulus characteristics, recording techniques, patient demographics and clinical grading systems of retinopathy severity. There is also some indication that the STR waveform is delayed with reduced amplitude in diabetic eyes [176].

Electrodiagnostic evaluation of neuronal function in retinal vascular occlusions is typically performed with the flash ERG as compromised retinal circulation affects retinal bipolar and ganglion cell function. The PR-VEP and PERG are not routinely employed in the clinical evaluation of retinal circulatory abnormalities as they are less informative in these conditions. Central Retinal Artery Occlusion, Central Retinal Vein Occlusion and Branch Retinal Vein Occlusion all reduce the amplitude of the b-wave and the PhNR [130, 177–188]. The PhNR is usually more reduced than the b-wave, implying greater dependence of more inner retinal neurones on retinal circulation [130, 177–183, 185–188]. The recovery of retinal function, whether spontaneously or as a result of treatment, is more robust with improvement b-wave in the initial phase and the PhNR recovering over longer time-period [130, 184, 186, 187]. The magnitude of PhNR amplitude reduction before treatment has been suggested to be a better predictor of visual prognosis than the b-wave [130, 179].

The non-perfusion or hypoperfusion of the optic nerve itself can result in an ischaemic optic neuropathy. Ischemic optic neuropathy (ION) typically comprises two major types; anterior (AION) which affects the optic nerve head, and posterior (PION) which affects the portion of the nerve posterior to the nerve head. These can be further characterised into those caused by an arteritic process or non-arteritic process (i.e. AAION or NAION respectively) [189]. The management of ION and their subtypes can differ, as can their visual prognosis [190].

The VEP abnormalities in ischaemic optic neuropathy were first formally introduced by Wilson [191] who demonstrated the amplitude reduction of the PR-VEP, with small or no changes in latency seen. Other studies are in general agreement with Wilson’s amplitude findings, but some studies report varying degrees of latency abnormality, suggesting commensurate conduction delay is not an exclusion to VEP diagnosis of ION [192–195]. Further, utilising a small check width PR-VEP was found to have higher sensitivity in the detection of optic nerve dysfunction [194]. There has been conflicting evidence to whether latency delay is a truly a feature of ION. Some of this may arise around the heterogeneous cohorts recruited within these early studies or consideration for the timing of PR-VEPs after the acute event. For example, it has since been noted that within the acute phase of ION that latency can be more affected [196]. Furthermore, it is unknown whether PR-VEP morphology changes or field defects were accounted for, as a central scotoma or altitudinal field defects can reduce the macular derived P100 and enhance the paramacular components of the VEP (i.e. producing a bifid or ‘p-n-p’ waveform). This can mistakenly be construed as an atypically early or delayed P100 as demonstrated by Thompson [197], therefore half-field PR-VEPs are essential to elaborate on the origins of the components observed, as are PR-VEPs to a range of check widths [198]. Overall, a fair conclusion now is that ION predominantly affects PR-VEP amplitude, but latency can in some circumstances be affected, but is typically less marked than that observed in optic neuritis [194, 197–202]. The flash VEP is typically lower amplitude with latency changes [202].

While the PERG N95 amplitude reduction is a common clinical feature in ischemic optic atrophy, this has been studied less frequently [194, 195, 201, 202]. Interestingly it was proposed that in the acute phase of visual loss, if the PERG is recorded within 8–35 days from onset this shows rapid loss of the N95 component in AION, whereas in optic neuritis this not as frequently seen [195, 203], but this finding has not been validated. PERG abnormalities of the P50 component have also been reported, which may suggest some incipient dysfunction upstream to the RGCs at the macular photoreceptors or bipolar cells, although this has not widely observed [195].

Rangaswamy et al. [129] demonstrated in 17 patients with NAION that the PhNR amplitude is reduced and its decrease correlated to the reduction in visual field sensitivity. Including a control group, these authors demonstrated in their Receiver Operating Curve that the PhNR has 96% diagnostic accuracy for AION. Interestingly it was also found that the PhNR was reduced in some degree in the asymptomatic eyes, suggesting there may be subtle signs of global RGC dysfunction before clinical signs of AION may appear. Further studies of the PhNR have included heterogenous cohorts of optic nerve disease, some including ischemic optic neuropathies, with variable degree of PhNR change [204, 205]. Beyond the PhNR, it has been shown that impairment of OPs can also occur in AION [206], which was observed particularly in OP2 and OP3 in a later study [202], presumably secondary to ischemic changes at the inner retina. The mfVEP can provide further information regarding optic nerve integrity, particularly as they demonstrate a close relationship between visual field loss and topographic mfVEP amplitude reduction, although this may overlap with other optic neuropathies [207, 208].

## Optic neuritis and demyelinating disease

Optic neuritis and other inflammatory disorders of the optic nerve can give rise to significant visual impairment, warranting early identification of aetiology which can facilitate diagnosis, prognosis and management. The use of electrophysiology in optic neuritis has been extensively studied in the context of demyelinating disorders. As the PR-VEP assesses the integrity of the visual pathway from the macula to striate cortex, it can be used in the detection of optic nerve and intracranial pathway abnormalities but can lack specificity for the underlying disease. For example, whilst optic neuritis is most historically associated with Multiple Sclerosis (MS), its presentation, history or PR-VEP changes can have wide differential diagnosis including infectious *(i.e. Lyme disease, Herpes Zoster, toxoplasmosis)*, vascular (*i.e. ION)*, toxic/nutritional *(i.e. B12 deficiency, ethambutol, or tobacco-alcohol toxicity)*, Compressive *(i.e. intracranial tumours)*, disseminated neurological disorder *(i.e. Adrenoleukodystrophy, Neuromyelitis Optica Spectrum (NMO) disorder*), hereditary optic neuropathies (i.e. LHON or ADOA) or systemic disease *(i.e. Systemic Lupis Erythematosus, Sjögrens syndrome or Sarcoid)* [209]. The type of PVEP abnormality may in some instances characterise some of these pathologies, but often demands the additional use of a PERG to further aid diagnosis.

Optic Neuritis can often be the first presentation of disease in MS, occurring in 13–15% of patients [210]. Furthermore, many patients with MS can show optic nerve or PR-VEP abnormalities despite the absence of visual symptoms or MRI abnormalities of the orbit [211–213]. The early works pioneering the clinical utility of the PR-VEP in optic neuritis came from Halliday et al. who demonstrated an increased latency in the PR-VEP as characteristic for conduction delay associated with demyelination [211, 214]. The PR-VEP in acute phase of optic neuritis is often unrecordable, significantly attenuated or delayed, likely subsequent upon conduction block of the axonal volley [215]. After the acute phase, amplitude often improves but the delay most commonly persists despite the resolution of visual acuity or field defects [211]. Over 6–36 months the latency can slowly decrease presumably concordant with remyelination, sodium channel reorganisation or cortical receptive field plasticity [216–218], but still remains abnormal in the majority of cases [219]. Amplitude recovery is more quickly and closely related with the clinical improvement in visual acuity [220]. It is important to note that in children normalisation of the PR-VEP is more common than in adults, often occurring within 12 months [221].

The abnormality of a PVEP should always be investigated with a PERG whenever possible to identify the locus of visual system dysfunction, as a PVEP abnormality may be consequent upon either a maculopathy or RGC dysfunction (Fig. 3). Holder [222] demonstrated that in 199 eyes with abnormal PVEPs from optic nerve demyelination, 39.2% of these demonstrated abnormality of the PERG, the majority of which affecting the N95 component. It was later demonstrated by Holder [79] in a large series that the reduction of the N95:P50 ratio of the PERG is closely related to a greater severity of conduction delay in the PR-VEP. It may take up to 6 weeks for a PERG abnormality to appear after acute optic neuritis. This reflects the time taken for retrograde degeneration to occur to RGC axons, which then predominantly affects the N95 component. It has been demonstrated that N95 component loss following optic neuritis is associated with a worse visual prognosis, and correlates with structural measures of macular ganglion cell complex volume (GCCV) and nerve fibre layer thickness [223–226]. In cases of severe N95 reduction, the P50 component may also reduce in amplitude and shorten in peak-time. In the acute phase, the P50 component can also be reduced reflecting some central retinal dysfunction, but this observation typically resolves within 4 weeks [79, 227]. As the PERG N95 loss in optic neuritis typically occurs after the acute phase of vision loss, a reduced PERG N95 during acute presentation suggests other primary RGC disease such as LHOA or other optic nerve pathology.

There are only a few studies at the time of writing that have investigated optic neuritis and demyelinating disease using the PhNR. So far, these suggest that the PhNR may be useful in the delineation between structural and functional visual measurements, where functional change can precede changes in RNFL thickness. Wang et al. [228] found that the PhNR in MS has reduced amplitude across an entire stimulus-intensity range tested, interestingly in eyes with and without a history of optic neuritis. Furthermore, in those with a history of optic neuritis, a close correlation can be observed to visual field changes and RNFL thickness. In particular, the most marked change observed was in patients with a history of optic neuritis longer than 6 months prior to testing, suggesting the PhNR is sensitive in detecting the retrograde degeneration of RGC cell axons. Whilst there may be some concern regarding the PhNR discriminatory ability against visual fields the only study to address this utilised reference data from two different populations and hence the verdict is inconclusive [228]. The PhNR during the acute phase of optic neuritis can also be abnormal, possibly secondary to neuronal swelling, inflammation or glial cell disruption which contribute to the PhNR [29, 92]. It has also been demonstrated using the focal macula ERGs, that the a- and b-waves were significantly reduced in the central 15° field alongside the PhNR reduction, which may persist following visual improvement [204]. It is interesting to note that the PhNR has shown earlier changes than structural measurements of RNFL thinning in severe inflammatory conditions causing optic atrophy, where the PhNR becomes abnormal after only 1 month whereas RNFL changes can take up to 3 months to manifest [229]. An illustration of a patient with optic neuritis is presented in Fig. 3 which shows the PERG, PR-VEP, F-VEP and PhNR waveform alterations.

The mfVEP can assess the functional regions of the optic nerve and therefore may be of benefit in some cases of optic neuritis. For example, in mild forms of optic neuritis, where topographically only a small segment of nerve axons are affected (i.e. causing a small scotoma or peripheral field defect), the conventional VEP may mask an underlying defect as it is produced from both normal and abnormally functioning axons, whereas the mfVEP may detect this focal loss [230, 231]. However, the technical demands of a mfVEP and its accessibility have limited its widespread use to date.

## Papilloedema and raised intracranial pressure

Raised intracranial pressure (rICP) and papilloedema can cause significant visual impairment if left untreated. The mechanism of visual loss is likely a result of RGC dysfunction secondary to mechanical or compressive effects on the optic nerve which disrupt axoplasmic flow resulting in nerve fibre swelling, clinically known as papilloedema [232]. As the effects of rICP impacts the RGCs, optic nerve and visual pathway, electrophysiology as an objective functional measure of these neuronal components offers advantages over existing methods such as visual acuity or papilloedema grading, which can be insensitive to change in rICP [233]. In particular, differentiation of optic disc oedema from rICP and from optic neuropathies, which is crucial for ongoing management, may be achieved through electrophysiology.

Early publications reported high correlation between N2 latency of the F-VEP and invasively recorded ICP, including reports that the F-VEP aided in the detection of posterior visual pathway abnormalities associated with paediatric rICP, such as ‘stretching’ the optic radiations or chiasmal dysfunction from ventricular enlargement or alteration [233–236]. However, the high inter-subject variability of the F-VEP has precluded any reliable absolute amplitude or latency measurements to indicate rICP and therefore its use in rICP is limited. Conversely, the PERG and PR-VEP demonstrate relatively low intra- and inter-subject variability. Early studies of PR-VEPs in rICP demonstrated P100 latency prolongation between patients with Idiopathic Intracranial Hypertension (IIH) and controls, with modest correlation with ICP. These PR-VEP alterations in normalised when papilloedema and blind-spot areas resolved [237, 238]. However, PR-VEPs in IIH have overall demonstrated variable sensitivity and clinical utility [239–241]. Nevertheless, an abnormal PR-VEP at baseline does predict worse visual outcome and shares a close relationship with visual field abnormalities [241, 242]. Other studies have demonstrated a high prevalence of PR-VEP abnormality in paediatric craniosynostosis [243], including the use of serial PR-VEPs in longitudinal monitoring of rICP, some demonstrating a 71% sensitivity and 100% specificity for rICP [244–246]. These were detected through longitudinal study of PR-VEPs recorded to a range of spatial frequencies, which improves the sensitivity of detecting rICP in craniosynostosis and IIH [247]. The variable sensitivity of PVEPs in these conditions likely reflects a variance of methodological approaches, such as only performing a PR-VEP to one spatial frequency or use of a small stimulus field size. The challenge with PR-VEPs as a marker of optic nerve and RGC dysfunction in IIH, is the site of dysfunction in IIH as a ‘pure’ form of rICP *(i.e. one where intracranial compliance is mostly unaffected, relative to Craniosynostosis or Hydrocephalus*) is predominantly at the RGCs, therefore the VEP as a measure of the entire visual pathway may conceal early changes, such as from mechanisms of post-retinal contrast adaptation [248, 249].

The PERG and PhNR hold promise in the objective measurement of visual function in patients with papilloedema and/or rICP. This can provide complementary topographic information of functioning RGC populations, which is of interest in rICP as the central visual field is often only affected late in the disease course [233]. It has been demonstrated that the steady state PERG can be abnormal in 77.7% of patients with IIH, particularly to higher spatial frequencies as observed in the PVEP [247]. The PERG N95 has a close relationship with VF sensitivity and OCT GCCV and RNFL thickness [250]. More recently, the PhNR was studied in IIH where they found 60% of patients to have reduced amplitude that correlated significantly with the SAP-MD and macula GCCV volume [251]. For one case in this cohort, aggressive ICP reducing treatment normalised the PhNR, improved the papilloedema grading, but did not alter the SAP-MD. This demonstrates some distinction from optic neuritis where the PhNR often remains abnormal despite resolution of visual symptoms. A later study compared the focal and full-field PhNR and PERG in patients with IIH, which corroborated their existing findings of an abnormal PhNR in IIH affecting the full-field more than focal PhNR, suggesting the topographic peripheral field abnormalities seen in IIH are concordant with the PhNR findings [252]. However, the comparative normality of the focal-PhNR compared to the abnormal PERG in the same patients are curious, perhaps reflecting differences between each stimulus modality and their relative origins. They did, however, find a relatively low sensitivity of the PERG in IIH (45.5%), perhaps reflecting differences between the transient and steady-state PERG and the influence of different spatial frequencies. These findings overall suggest that the PhNR and PERG are useful indicators of optic nerve and RGC function in rICP, but to date have not been directly associated with ICP measurements as a surrogate marker.

## Visual electrophysiology in practice

**Fig. 5:**
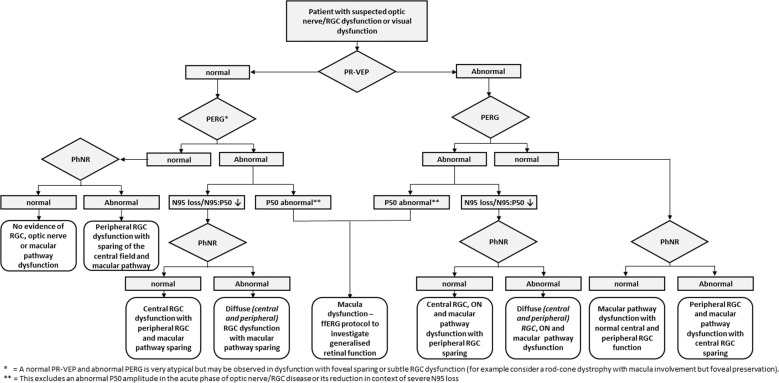
Proposed diagnostic algorithm for the use of the pattern electroretinogram (PERG), pattern visual evoked potential (PVEP) and photopic negative response (PhNR) in clinical practice. The reader can follow the different scenarios whereby the PVEP, PERG or PhNR may be normal or abnormal, to ascertain what clinical conclusions may be drawn from these findings. It is stressed by the authors, that this algorithm should be interpreted in the clinical context, and not all diseases or patterns of optic nerve or retinal ganglion cell dysfunction conform to these patterns. In these circumstances, detailed clinical examination, history taking, imaging, genetic or other laboratory testing may be necessary to aid diagnosis.

In this review we have studied a variety of techniques, such as the PERG, VEP and PhNR, in conditions affecting the optic nerve and RGCs. The authors encourage those undertaking electrophysiological testing to meet the minimum standards for recording and exceed these where possible, to further characterise the site of dysfunction. In the clinical interpretation of data, one may wish to interpret findings of PERGs, VEPs and PhNR together to aid decision making and analysis. As such, we propose a simple decision flow-chart for the interested reader in how to interpret these tests together, to aid clinical interpretation of electrophysiological tests (Fig. 5). It is important to consider the clinical and technical factors which may influence findings, such as test time after an acute event, co-morbidities or reduced compliance. The authors encourage recording a PERG and PR-VEP simultaneously, where possible, to maximise the diagnostic value of electrophysiological tests alongside factoring in these variables.

## Conclusion

Visual electrophysiology is a key diagnostic tool in the assessment of conditions affecting RGCs and the optic nerve. In this review we have discussed the role of the PERG, VEP, PhNR in characterising visual function in RGC and optic nerve disease. The benefits of electrophysiology are to provide functional data of the visual system to complement structural data and the clinical examination. At the advent of the genomic era and beginning of new exciting therapies for optic nerve disease, functional measurements will be essential for measuring safety, efficacy and outcome. Furthermore, the objective nature of electrophysiology testing means information about the visual system can be gained from patients unable to complete subjective tests. The ability to assess RGC and optic nerve function quantitatively and through different but complementary tests builds a diagnostic platform for phenotyping of disease, which can aid in clinical decision making.
