# Correction: Clinical electrophysiology of the optic nerve and retinal ganglion cells

**DOI:** 10.1038/s41433-021-01798-2

**Published:** 2021-10-13

**Authors:** Oliver R. Marmoy, Suresh Viswanathan

**Affiliations:** 1 Clinical and Academic Department of Ophthalmology, Great Ormond Street Hospital for Children, London, UK; 2 UCL-GOS Institute for Child Health, University College London, London, UK; 3 Manchester Metropolitan University, Manchester, UK; 4 College of Optometry, State University of New York, New York, NY USA

**Keywords:** Optic nerve diseases, Retina

Correction to: *Eye* 10.1038/s41433-021-01614-x, published online 11 June 2021

Figure 1 illustration had an error—in the second panel (the PhNR waveform), the ‘a’ and ‘b’ labels were the wrong way around.

The original article has been corrected

